# Rett mutations attenuate phase separation of MeCP2

**DOI:** 10.1038/s41421-020-0172-0

**Published:** 2020-06-16

**Authors:** Chunyan Fan, Honglian Zhang, Liangzheng Fu, Yuejiao Li, Yi Du, Zilong Qiu, Falong Lu

**Affiliations:** 1 State Key Laboratory of Molecular Developmental Biology, Institute of Genetics and Developmental Biology, Chinese Academy of Sciences, 100101 Beijing, China; 2 University of Chinese Academy of Sciences, 100049 Beijing, China; 3 Chinese Academy of Sciences Center for Excellence in Brain Science and Intelligence Technology, Chinese Academy of Sciences, 200031 Shanghai, China; 4 State Key Laboratory of Neuroscience, Institute of Neuroscience, Chinese Academy of Sciences, 200031 Shanghai, China; 5 The Innovative Academy of Seed Design, Chinese Academy of Sciences, 100101 Beijing, China

**Keywords:** DNA methylation, Chromatin

Dear Editor,

Methyl-CpG-binding protein 2 (MeCP2) is a ubiquitously expressed nuclear protein originally identified as a methylated DNA binding protein, which is particularly abundant in mature neurons^1,2^. Deficiency or excess of MeCP2 causes severe neurological problems. Mutations in MeCP2 account for 95% of the dominant X-linked neurological disorder Rett syndrome^3^. MeCP2 have two key functional domains: the methyl-DNA binding domain (MBD) and the transcriptional repressor domain (TRD). Almost all of missense Rett mutations are clustered in these two domains, such as R133C, F155S, T158M in MBD, and R306H in TRD^4,5^. The mechanism of the mutations leading to Rett syndrome is still not well understood. Here, we reveal that MeCP2 can drive the liquid–liquid phase separation (LLPS) in complex with DNA. Interestingly, this ability is compromised in the presence of mutations found in Rett syndrome patients, suggesting a potential common mechanism by disrupting LLPS of MeCP2 droplets underlying Rett syndrome.

MeCP2 forms sharp condensed foci which highly overlap with DNA dense compartments in neuronal nuclei^6,7^. Recently, LLPS has been recognized as an important mechanism to condensate molecules to form membraneless compartments within a cell^8^. As both the MBD and TRD of MeCP2 bind to DNA^9^, we hypothesized that the sharp puncta of MeCP2 in the nuclei were phase separated liquid droplets mediated by multivalent interactions between MeCP2 and DNA. To test this hypothesis, we purified full-length recombinant His-MBP-MeCP2 (mouse MeCP2-e2 if not specified) and released the His-MBP tag by cutting with TEV protease (Supplementary Fig. S1). CpG methylated DNA was prepared by methylating a 1.1 kb DNA fragment with the M.SssI methyltransferase and verified by digesting with a methylation sensitive endonuclease, BsiWI (Supplementary Fig. S2). Phase separation was assayed using different concentration of a 1.1 kb unmodified or methylated DNA with MeCP2 in a buffer containing 150 mM NaCl and a low concentration of DAPI for visualizing DNA. Puncta formation was observed both in the presence of DNA or methylated DNA (Fig. 1a). In contrast, MeCP2 protein alone and DNA alone could not form the puncta (Supplementary Fig. S3a, b). In addition, His-MBP in mixture with mEGFP protein with or without DNA could not form puncta either (Supplementary Fig. S3c), confirming that MeCP2 together with DNA or methylated DNA drives the puncta formation.

**Fig. 1:**
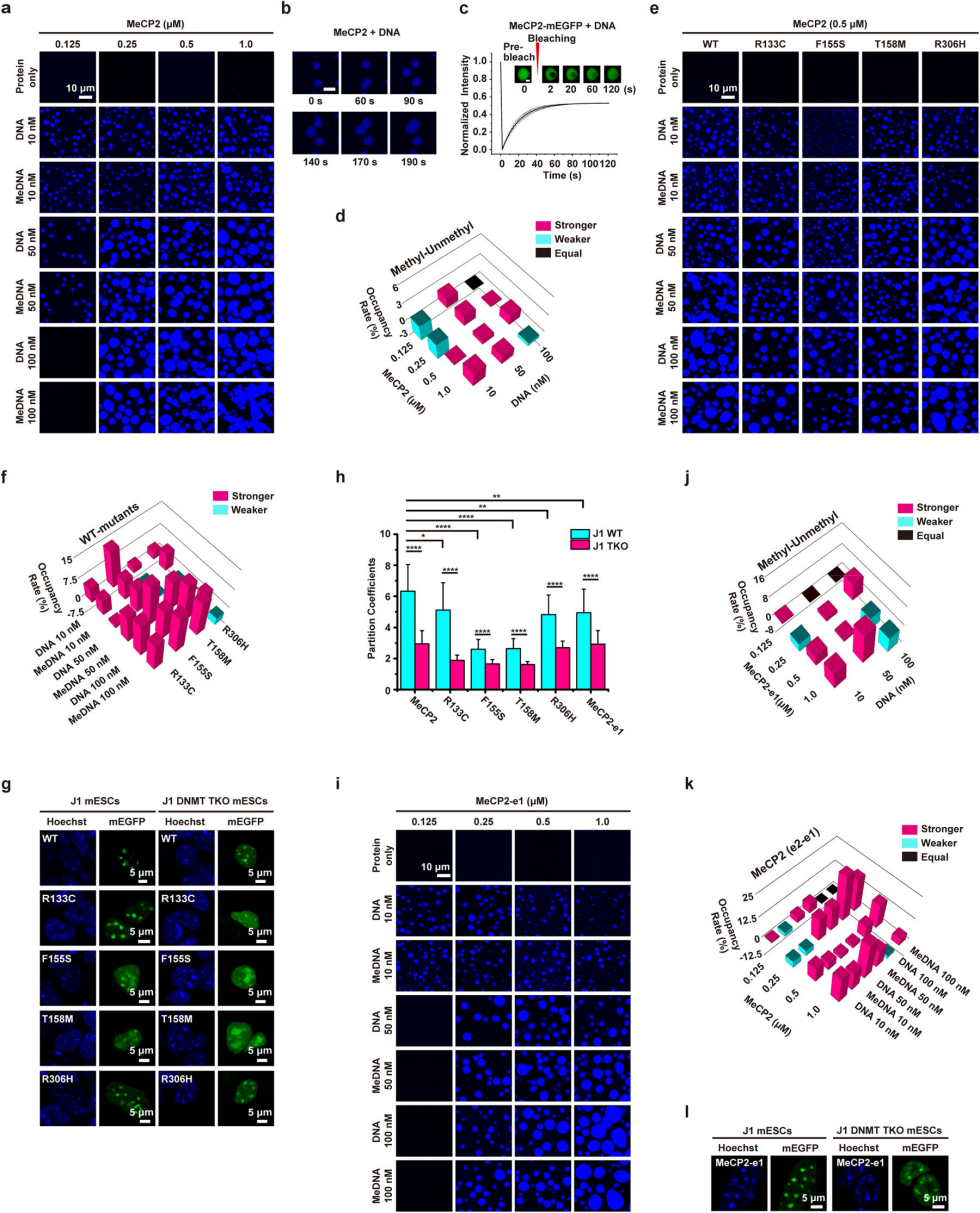
Rett mutants attenuate phase separation of MeCP2. **a** Phase diagram of MeCP2 with unmethylated DNA or methylated DNA (MeDNA) in 150 mM NaCl. DAPI was used for DNA visualization. Scale bar, 10 µm. **b** Fusion of droplets formed by MeCP2 protein (1.0 μM) with DNA (100 nM) in 150 mM NaCl. Scale bar, 5 µm. **c** FRAP analysis of droplets formed by MeCP2-mEGFP protein (1.0 μM) with DNA (100 nM) after incubating 5 min on 384-well microplates. Scale bar, 2 µm. All data are presented as mean ± SD (*n* = 3). **d** Comparison of phase diagrams of MeCP2 with methylated DNA (MeDNA) and unmethylated DNA in Fig. 1a. Pink, cyan, and black squares indicate stronger, weaker and unchanged LLPS. **e** Phase diagram of MeCP2 wild-type and Rett mutant proteins (0.5 µM) with DNA or MeDNA in 150 mM NaCl. DAPI was used for DNA visualization. Scale bar, 10 µm. **f** Comparison of phase diagrams of MeCP2 wild-type and mutant proteins (0.5 µM) with DNA or MeDNA in (**e**). **g** Subcellular localization of MeCP2 and Rett mutants tagged with mEGFP overexpressed in J1 wild-type and DNMT TKO mESCs. Hoechst was used to stain DNA. Scale bar, 5 µm. **h** Partitioning quantification of MeCP2-mEGFP proteins. ****p < 0.0001, ***p < 0.001, **p < 0.01, *p < 0.1. **i** Phase diagram of MeCP2-e1 with DNA or MeDNA in 150 mM NaCl. DAPI was used for DNA visualization. Scale bar, 10 µm. **j** Comparison of phase diagrams of MeCP2-e1 with MeDNA and DNA in (**i**). **k** Comparison of phase diagrams of MeCP2-e2 and MeCP2-e1 with DNA or MeDNA in (**a**, **i**). **l** Subcellular localization of MeCP2-e1-mEGFP overexpressed in J1 wild-type and DNMT TKO mESCs. Scale bar, 5 µm.

Next, we monitored the property of the puncta in more details. The MeCP2/DNA droplets can fuse together upon contact (Fig. 1b). Moreover, we observed quick recovery of EGFP fluorescence after photo-bleaching of the middle of MeCP2-mEGFP/DNA droplets (Fig. 1c). These evidences support that the puncta formed by MeCP2 and DNA are LLPS droplets. To compare the ability of DNA and methylated DNA in promoting the phase separation of MeCP2, we calculated ‘Occupancy Rate’ of the droplets formed by MeCP2 with methylated DNA over unmethylated DNA. We found that about 66% values were positive (stronger) and 25% values were negative (weaker), indicating that CpG methylation could moderately promote LLPS of MeCP2 in complex with DNA (Fig. 1d). Taken together, these data reveal that MeCP2 can undergo LLPS with DNA which can be moderately enhanced by methylation at CpG sites.

Rett mutations have been found both within and outside the MBD domain^4^. Therefore, the loss of the binding to methylated CpG is not the sole mechanism of MeCP2 mutations in causing Rett syndrome. We asked whether the phase separation impairment can be a common result of Rett mutations. We examined the effect of three missense mutations in the MBD domain (R133C, F155S and T158M) and one in the TRD domain (R306H) (Supplementary Fig. S4). MeCP2 proteins with the above Rett mutations were mixed with DNA or methylated DNA to explore whether Rett mutations affect the phase separation of MeCP2. We found that the phase separation of these mutants was weaker than that of the wild-type protein under the same conditions (Fig. 1e, f, Supplementary Fig. S3d), indicating that the Rett mutations attenuate phase separation of MeCP2 in vitro.

In order to study whether Rett mutations affect the condensation of MeCP2 in vivo, we transfected wild-type MeCP2 and Rett mutants tagged with mEGFP to J1 mESCs (mouse embryonic stem cells). Western blot with MeCP2 antibody showed that the exogenous MeCP2-mEGFP level was slightly higher than the endogenous MeCP2 level in J1 mESCs, but lower than that in adult mouse brain sample (Supplementary Fig. S5), indicating that the MeCP2-mEGFP level was within the range of physiological level. MeCP2 formed sharp puncta preferentially associated with Hoechst-dense foci in vivo (Fig. 1g), which was consistent with previous studies^6,7^. F155S and T158M mutants were partially excluded from Hoechst-dense region and mis-localized to nucleoli similar to previous observation of a MBD domain deletion mutant^7^. The R133C and R306H mutants remained mainly in Hoechst-dense region (Fig. 1g). FRAP analysis of the puncta indicated that the mobility of the MeCP2 within puncta was enhanced in the presence of Rett mutations (Supplementary Fig. S6, Table S1). Quantifying the condensation of MeCP2 and the mutants using ‘partition coefficient’ approach^10^, the puncta of the Rett mutants showed obvious reduced condensation compared to WT protein (Fig. 1h), indicating that Rett mutations impaired the condensation of MeCP2 in vivo.

To test whether DNA methylation further affect the puncta of MeCP2 in vivo, we took advantage of J1 DNMT TKO (*Dnmt1*^*−/−*^*Dnmt3a*^*−/*−^*Dnmt3b*^*−/−*^) mESCs in which all the major DNA methyltransferases (DNMT1, DNMT3A, and DNMT3B) were depleted, while the chromatin structure was minimally affected^11,12^. MeCP2 wild-type, R133C and R306H still formed puncta at Hoechst-dense foci in J1 DNMT TKO mESCs, while F155S and T158M mutants were mostly excluded from Hoechst-dense region (Fig. 1g). FRAP analysis showed the mobility of MeCP2 was enhanced in DNMT TKO cells compared to that in WT cells (Supplementary Fig. S7, Table S1). Compared to puncta seen in wild-type mESCs, the condensation of MeCP2 and Rett mutants expressed in DNMT TKO mESCs were weakened (Fig. 1g, h). These data are consistent with the in vitro results that methylated DNA likely further enhances LLPS of MeCP2 compared to unmodified DNA. We noted that DNA methylation showed larger impact on the condensation of MeCP2 in vivo than in vitro (Fig. 1a, d, g, h), suggesting additional regulation of MeCP2 condensation in vivo. Together, these data show that DNA methylation can enhance the condensation of MeCP2 in vivo.

The MeCP2 gene gives rise to two different isoforms with different N-terminals due to alternative inclusion of exon 2 in e2 isoform (MeCP2-e1 and MeCP2-e2)^6^ (Supplementary Fig. S4). We asked if MeCP2-e1 was with different ability in driving LLPS as MeCP2-e2. We found that DNA binding could drive MeCP2-e1 to form LLPS, while CpG methylation could slightly enhance the LLPS (Fig. 1i, j, Supplementary Fig. S3e). Interestingly, the phase separation of MeCP2-e1 was moderately weaker than that of MeCP2-e2 (Fig. 1k). MeCP2-e1 tagged with mEGFP formed puncta in mESCs similarly to MeCP2-e2, but with weaker condensation and higher mobility (Fig. 1g, h, l, Supplementary Fig. S8, Table S1). The different ability of the two MeCP2 isoforms in driving LLPS may be related to the difference of their DNA binding affinity^13^, suggesting functional divergence of the two isoforms which warrants future study.

In this study, we reveal that MeCP2 together with DNA can drive LLPS, which can be moderately enhanced by the presence of CpG methylation. Interestingly, the two isoforms show different capability in driving LLPS. More interestingly, this ability of MeCP2 in driving LLPS is commonly compromised in the presence of mutations found in Rett syndrome patients both inside and outside MBD domain. A recent report published during revision of this study also revealed the impairment of MeCP2 phase separation by Rett mutations^14^. These findings suggest a potential role of MeCP2 in driving the formation of chromatin compartmentation necessary for normal neuronal function, providing a new angle to understand the mechanism underlying Rett syndrome.

## Supplementary information

Supplementary Information

## Supplementary information

**Supplementary Information** accompanies the paper at (10.1038/s41421-020-0172-0).
