# Supplementary material for: Rett mutations attenuate phase separation of MeCP2
Source: Cell Discov. 2020 Jun 16;6:38. doi: 10.1038/s41421-020-0172-0 (PMC7296026; doi:10.1038/s41421-020-0172-0)
Supplement: Supplementary file 1 — Supplementary Information [file 41421_2020_172_MOESM1_ESM.pdf]

Rett Mutations Attenuate Phase Separation of MeCP2

Fan*, et al.*

Supplemental Information

The supplementary information consists of 8 supplementary figures, 1 supplementary table and methods.

SUPPLEMENTAL FIGURES

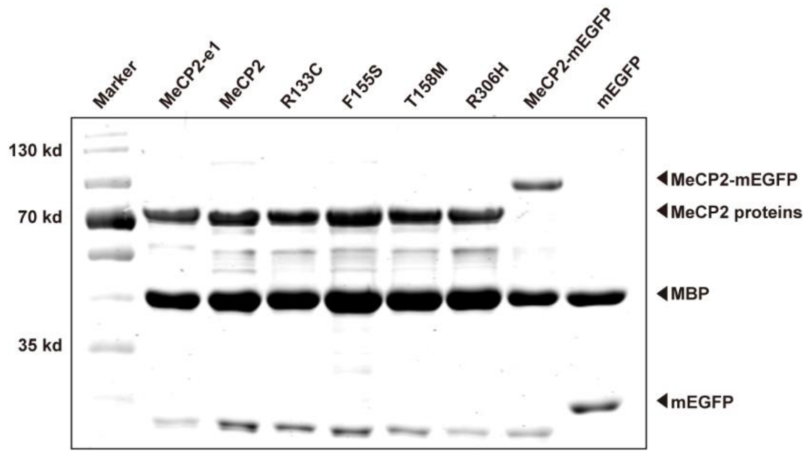

Supplemental Figure S1. SDS-PAGE of the recombinant proteins used in this study.

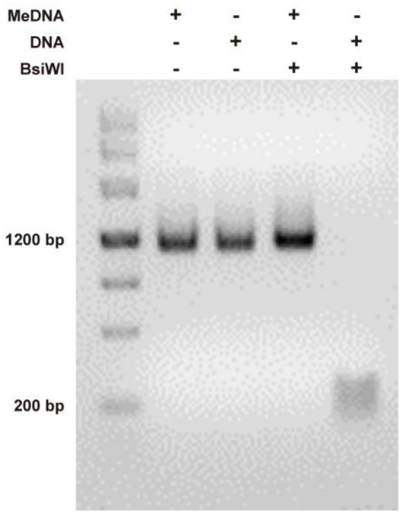

Supplemental Figure S2. Validation of the methylation status of the methylated DNA by digestion with methylation sensitive BsiWI.

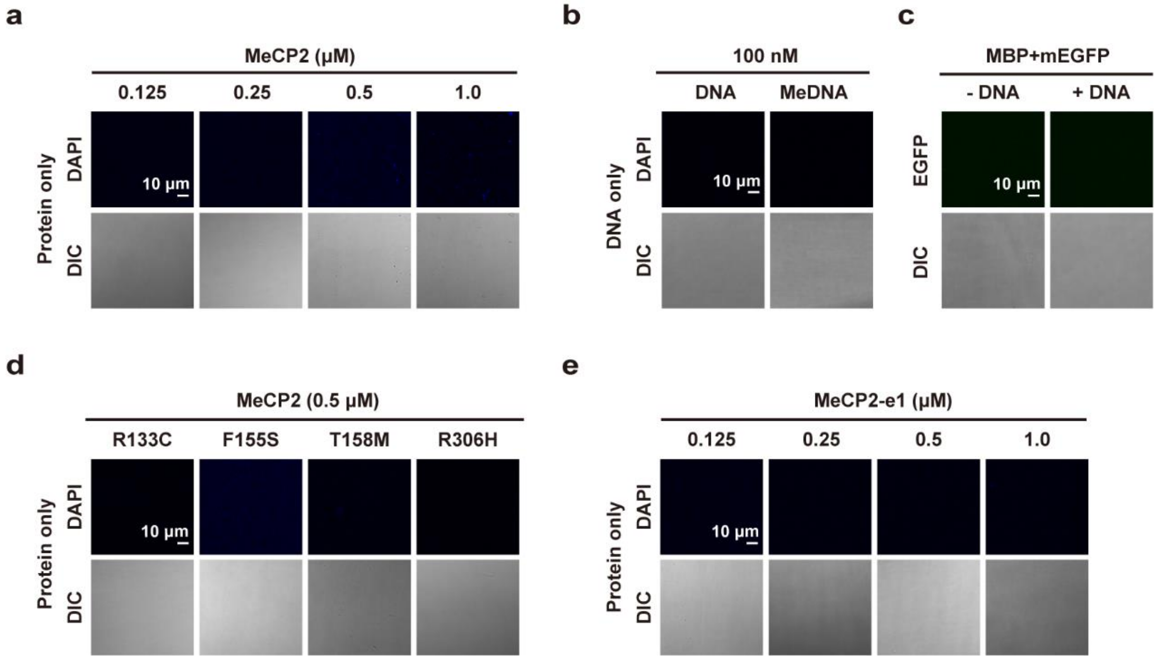

Supplemental Figure S3. MeCP2 and DNA alone cannot drive phase separation in the buffer containing 150 mM NaCl. (**a**, **d**, **e**) MeCP2 proteins without DNA. (**b**) DNA (100 nM) or MeDNA (100 nM) only. (**c**) His-MBP in mixture with mEGFP protein (1.0 μM) only (left panel) or with100 nM DNA (right panel). Scale bar, 10 μm.

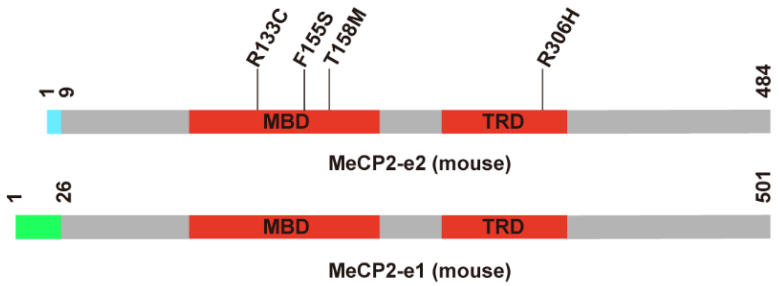

Supplemental Figure S4. The diagram of MeCP2 (-e1, -e2 and mutants) proteins analyzed in this study.

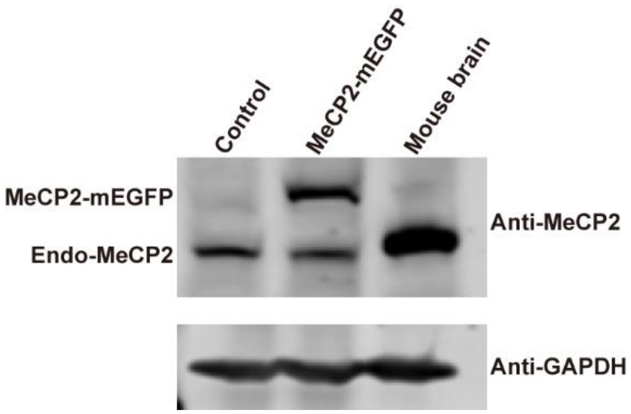

Supplemental Figure S5. Western blot analysis to check the protein level of overexpressed MeCP2-mEGFP and endogenous MeCP2 in J1 mESCs and in adult mouse brain tissues.

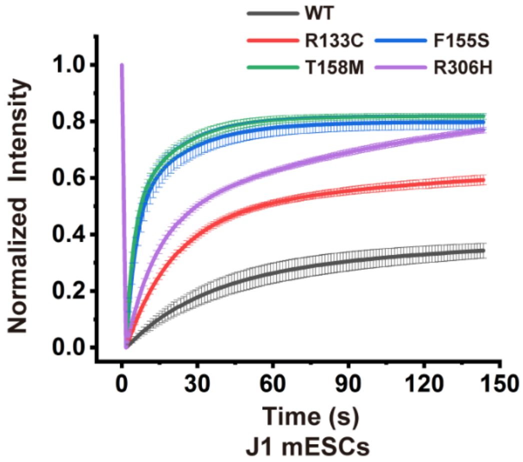

Supplemental Figure S6. The FRAP analysis of puncta of MeCP2 WT or Rett-related mutants in J1 wild-type mESCs. FRAP was performed at Hoechst-dense region (n=3).

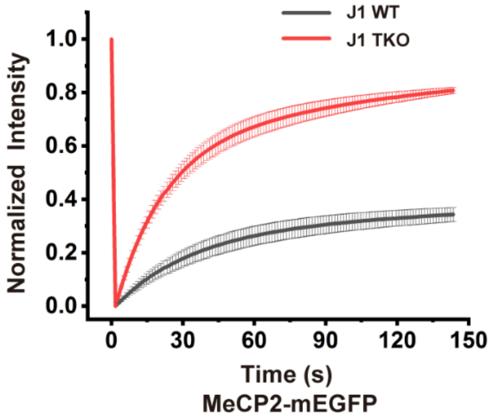

Supplemental Figure S7. The FRAP analysis of MeCP2 puncta in J1 wild-type and DNMT TKO mESCs. FRAP was performed at Hoechst-dense region (n=3). The FRAP data of MeCP2-mEGFP in J1 WT cells (black curve) was the same as the black curve in Supplemental Figure S6.

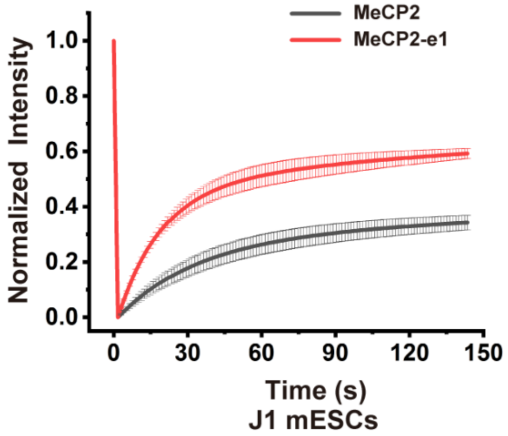

Supplemental Figure S8. The FRAP analysis of MeCP2 and MeCP2-e1 puncta in J1 mESCs. FRAP was performed at Hoechst-dense region (n=3). The FRAP data of MeCP2 in J1 mESCs (black curve) was the same as the black curve in Supplemental Figure S6.

Supplemental Table S1. FRAP Recovery Times, Modeled by a Double-Exponential Equation. Related to Supplemental Figure 6, 7 and 8.

Cells	τ 1(s)	τ 2(s)	Average τ(s)	Related to
J1-MeCP2	30.33	183.98	53.86	Supplemental Figure S6
J1-R133C	18.08	112.67	35.41	Supplemental Figure S6
J1-F155S	3.80	21.44	7.18	Supplemental Figure S6
J1-T158M	2.87	17.70	5.24	Supplemental Figure S6
J1-R306H	11.38	111.20	20.12	Supplemental Figure S6
J1 TKO-MeCP2	19.18	130.29	39.76	Supplemental Figure S7
J1-MeCP2-e1	16.37	317.21	32.86	Supplemental Figure S8

METHODS

Plasmids

Mouse MeCP2-e1, MeCP2-e2, MeCP2-e2-mEGFP and mEGFP were cloned into *E. coli* expression plasmid pMCSG7 vectors, with a 6xHis tag and a Maltose-binding protein (MBP) at the N-terminus followed by a tobacco etch virus (TEV) protease cleavage site motif. Mouse MeCP2-e1, MeCP2-e2 were inserted into the eukaryotic expression plasmid pmEGFP-C1 for experiments within cells. MeCP2 Rett mutant plasmids were cloned by site-directed mutagenesis strategy and recombined with NEBuilder (NEB, E2621). Mutations were confirmed by Sanger sequencing.

Expression, and purification of recombinant MeCP2

The recombinant bacterial expression vectors were transformed into *E. coli* BL21 (DE3). The cells were cultured at 37 ℃ in LB with 100 ug/mL ampicillin until OD 600 about 0.8. The protein expression was induced by adding IPTG to a final concentration of 0.1 mM at 16 ℃ overnight. The cells were collected by centrifugation at 5000 g, and then resuspended in lysis buffer (20 mM NaH_2PO_4, 0.5 M NaCl, 20 mM imidazole, 5% Glycerol, pH 7.4). The cells were lysed by sonication. The *E. coli* DNA was removed by precipitation with 0.2% PEI in the total extract. After removing the pellet containing DNA, the supernatant was further treated with 40% ammonium sulfate to precipitate recombinant MeCP2 protein. Then the pellet was dissolved in lysis buffer and used for affinity purification by Ni-NTA Agarose (Thermo Fisher Scientific). All the purified recombinant proteins were digested by TEV protease at 4 ℃ for more than 12 hours to remove the MBP tag. The proteins were dialyzed and stored in storage buffer (50 mM Tris-HCl, 500 mM NaCl, 0.5 mM EDTA, 1 mM DTT, 5% Glycerol, pH 8.0) at -80 ℃.

Preparation of unmodified and methylated DNA

DNA and methylated DNA were prepared as following. Both DNA used in experiment was 6 × 601 tandem repeats of 1122 bp in length. The sequence of the 601 bp DNA repeats is listed as described previously[1]:

CCGGATCCCCTGGAGAATCCCGGTGCCGAGGCCGCTCAATTGGTCGTAGACAG
CTCTAGCACCGCTTAAACGCACGTACGCGCTGTCCCCCGCGTTTTAACCGCCA
AGGGGATTACTCCCTAGTCTCCAGGCACGTGTCACATATATACATCCTGTTCCA
GTGCCGG

The 6 × 601 DNA from pWM530 vector was digested by restriction endonuclease BtgI (R0608S, NEB) at 37 ℃ overnight and gel extracted using FastPure Gel DNA Extraction Mini Kit (DC301-01, Vazyme). DNA was dissolved in nuclease free water. DNA was methylated by M.SssI (M0226V, NEB). 1 μg DNA was methylated in system containing 10 mM Tris-HCl, 50 mM NaCl, 10 mM $MgCl_2$, 1 mM DTT, 160 μM SAM and 4 units of M.SssI at 37 ℃ for 4 hours. After reaction, the methylated DNA was purified using phenol-chloroform extraction and dissolved in nuclease free water. Methylation-sensitive endonucleases BsiWI-HF (NEB #R3553) was used for further verification of the methylation status. 100 ng methylated DNA was digested with 10 U BsiWI-HF at 37 ℃ for 3 hours.

Phase separation assays *in vitro*

In vitro phase separation experiments was performed in 25 mM Tris-HCl pH 7.5, 1 mM DTT with 150 mM NaCl, at various protein concentrations (0.125 μM, 0.25 μM, 0.5 μM and 1.0 μM) and different concentration (0 nM, 10 nM, 50 nM and 100 nM) of DNA or methylated DNA. For imaging, assays were performed on 384-well microplates (CellCarrier, 6057300) and confocal imaging was carried out with Olympus FV1200 microscopy. All the data was analyzed by ImageJ and OriginPro.

Cells culture and transfection

J1 WT and DNMT TKO mESCs were cultured in DMEM (Life Technologies) supplemented with 15% fetal bovine serum, penicillin/streptomycin, nonessential amino acid, sodium pyruvate, GlutaMax, b-mercaptoethanol, and 1000 U/mL LIF (Millipore, ESGRO). Cells were seeded in a confocal dish (Cellvis, D35-20-1-N) for imaging. When the confluence of cells reached 70%, mEGFP-tagged MeCP2 and mutants plasmids were

transfected by Lipo3000 (Life, 23966). The final concentration of plasmids was 0.3 μg/mL. Living cells were imaged with an Olympus FV1200 confocal microscopy 24 h after transfection.

Western blot

Cells with MeCP2-mEGFP transfection were collected 48 hours after transfection. EGFP positive cells were sorted by flow cytometry (BD FACSAria III). Samples of the control cell and mEGFP positive cells were made by boiling the cells in 1xSDS loading buffer for 5 minutes. Mouse brain tissues were separated from adult (6~8 weeks) mice. The tissues were lysed by RIPA lysis buffer with protease inhibitor and boiled in SDS loading buffer. The samples were separated by SDS-PAGE and transferred to nitrocellulose. The membranes were blocked with blocking buffer at RT for 1 hour, and incubated with primary antibodies (rabbit anti-MeCP2 ,1:2,000, Millipore, 07-013; mouse anti-GAPDH, 1:10,000, Proteintech, 60004-1-Ig) overnight at 4 °C. After washing with TBST, the membranes were incubated with secondary antibodies (IRDye 680RD goat anti-rabbit, 1:10,000, LI-COR, 925-68071; IRDye 800CW goat anti-mouse, 1:10,000, LI-COR, 925-32210) for 1 hour at RT followed by washing. The images were taken by LI-COR Odyssey CLX imaging system.

FRAP measurements

An Olympus FV1200 microscope equipped with a 60 × oil objective was used for FRAP imaging. Defined regions were photo bleached at 488 nm laser (100% intensity, dwell time 500 ms). The fluorescence intensities in these regions were collected every 1.1 s and normalized to the initial intensity before bleaching. Each curve is an average of FRAP events from three distinct puncta and each data point is representative of the mean and standard deviation of fluorescence intensities. All FRAP experiments were conducted under the same conditions. FRAP data was analyzed by cellSens and OriginPro.

Quantification and statistical analysis

Images of 317*317 μm^2 were used for the calculation of 'Occupied Rate'[2,3] *in vitro*, and

thresholding method was used for droplet segmentation. 'Partition coefficient'[4,5] (fluorescence signals of MeCP2-mEGFP$_{droplet}$/MeCP2-mEGFP$_{bulk}$) in more than 20 cells were calculated in each of the analysis *in vivo*. MeCP2-mEGFP$_{droplet}$ means the fluorescence signals at Hoechst-dense region (droplet phase) and MeCP2-mEGFP$_{bulk}$ means the fluorescence signals at Hoechst-dispersed region (bulk phase). Statistical analyses were carried out using ImageJ, Excel, cellSens and OriginPro. Data are presented as mean ± SD.

References:

1 Song, F. *et al.* Cryo-EM study of the chromatin fiber reveals a double helix twisted by tetranucleosomal units. *Science* **344**, 376-380, doi:10.1126/science.1251413 (2014).

2 Wang, L. *et al.* Histone Modifications Regulate Chromatin Compartmentalization by Contributing to a Phase Separation Mechanism. *Mol Cell* **76**, 646-659 e646, doi:10.1016/j.molcel.2019.08.019 (2019).

3 Wang, L. *et al.* Rett syndrome-causing mutations compromise MeCP2-mediated liquid-liquid phase separation of chromatin. *Cell Res*, doi:10.1038/s41422-020-0288-7 (2020).

4 Pak, C. W. *et al.* Sequence Determinants of Intracellular Phase Separation by Complex Coacervation of a Disordered Protein. *Mol Cell* **63**, 72-85, doi:10.1016/j.molcel.2016.05.042 (2016).

5 Beutel, O., Maraspini, R., Pombo-Garcia, K., Martin-Lemaitre, C. & Honigmann, A. Phase Separation of Zonula Occludens Proteins Drives Formation of Tight Junctions. *Cell* **179**, 923-936 e911, doi:10.1016/j.cell.2019.10.011 (2019).
